# Generalized Kinetic Equations with Fractional Time-Derivative and Nonlinear Diffusion: H-Theorem and Entropy

**DOI:** 10.3390/e26080673

**Published:** 2024-08-08

**Authors:** Ervin K. Lenzi, Michely P. Rosseto, Derik W. Gryczak, Luiz R. Evangelista, Luciano R. da Silva, Marcelo K. Lenzi, Rafael S. Zola

**Affiliations:** 1 Departamento de Física, Universidade Estadual de Ponta Grossa, Ponta Grossa 84030-900, PR, Brazil; michelyrosseto@gmail.com; 2 National Institute of Science and Technology for Complex Systems, Centro Brasileiro de Pesquisas Físicas, Rio de Janeiro 22290-180, RJ, Brazil; luciano@fisica.ufrn.br; 3 Independent Researcher, Irati 84507-012, PR, Brazil; derikwilliam.gryczak@gmail.com; 4 Departamento de Física, Universidade Estadual de Maringá, Maringá 87020-900, PR, Brazil; lre@dfi.uem.br; 5 Istituto dei Sistemi Complessi (ISC–CNR), Via dei Taurini, 19, 00185 Rome, Italy; 6 Department of Molecular Science and Nanosystems, Ca’Foscari University of Venice, Via Torino, 155, 30172 Mestre, Italy; 7 Departamento de Física, Universidade Federal do Rio Grande do Norte, Natal 59078-900, RN, Brazil; 8 Departamento de Engenharia Química, Universidade Federal do Paraná, Curitiba 80060-000, PR, Brazil; lenzi@ufpr.br; 9 Department of Physics, Universidade Tecnológica Federal do Paraná, Apucarana 86812-460, PR, Brazil; rzola1@kent.edu

**Keywords:** entropy, nonlinear diffusion, anomalous diffusion, H-theorem

## Abstract

We investigate the H-theorem for a class of generalized kinetic equations with fractional time-derivative, hyperbolic term, and nonlinear diffusion. When the H-theorem is satisfied, we demonstrate that different entropic forms may emerge due to the equation’s nonlinearity. We obtain the entropy production related to these entropies and show that its form remains invariant. Furthermore, we investigate some behaviors for these equations from both numerical and analytical perspectives, showing a large class of behaviors connected with anomalous diffusion and their effects on entropy.

## 1. Introduction

After the seminal contributions of Boltzmann [1], Maxwell [2,3], and Gibbs [4] (see also Ref. [5]), the kinetic equations have emerged as fundamental tools for characterizing the dynamics of microscopic particles [6,7] and their interplay with observable thermodynamic phenomena. These equations find application across various domains, encompassing both equilibrium and non-equilibrium statistical mechanics frameworks, where they play a crucial role in extracting valuable insights about a system’s behavior from the underlying microscopic dynamics. Particularly significant is the pursuit of a functional description of the probability density whose temporal evolution exhibits a definite minus sign known as the H-theorem. This theorem serves as a critical characteristic for irreversibility in the system’s evolution, akin to the second law of thermodynamics, thereby shedding light on the emergence of macroscopic thermodynamic quantities from microscopic considerations. This function is directly connected with entropy, a crucial ingredient of this theory, establishing that non-equilibrium systems will reach equilibrium after long-time evolution. The H-theorem is also a way of investigating the rule of additivity for systems with different entropies, as discussed in Refs. [8,9,10,11].

The scenarios belonging to non-equilibrium statistical mechanics may be analyzed by different approaches, particularly Fokker–Planck-like equations. In linear form, the Fokker–Planck equation can be written as follows:

(1) $$\begin{array}{c} \frac{\partial }{\partial t}\rho \left(x,t\right)=\frac{\partial }{\partial x}\left\{D\frac{\partial }{\partial x}\rho \left(x,t\right)-F\left(x,t\right)\rho \left(x,t\right)\right\}\;, \end{array}$$

where ρ(x,t) is the distribution function and *D* is the diffusion coefficient. In the absence of external forces, F(x,t), the solution corresponds to the Gaussian distributions. The fractional Fokker–Planck equations, incorporating fractional operators, are usually written as follows:

(2) $$\begin{array}{c} \tau_{\gamma }\int_{0}^{t}dt^{\prime }K_{\gamma }\left(t-t^{\prime }\right)\frac{\partial }{\partial t^{\prime }}\rho \left(x,t^{\prime }\right)=\frac{\partial }{\partial x}\left\{D\frac{\partial }{\partial x}\rho \left(x,t\right)-F\left(x,t\right)\rho \left(x,t\right)\right\}\;, \end{array}$$

where $\tau_{\gamma }$ is a relaxation time and $K_{\gamma }\left(t\right)$ is a kernel related to the memory effects, which is connected to non-Debye relaxation processes [12,13]. The fractional operator can be related to different scenarios, as described in Refs. [14,15,16,17]. Equation (2) has been successfully applied in different contexts such as electrical impedance [12], anomalous transport in biological cells [18], anomalous diffusion in crowded environments [19], and in bioengineering [20]. We may also have the nonlinear Fokker–Planck equations, such as:

(3) $$\begin{array}{c} \frac{\partial }{\partial t}\rho \left(x,t\right)=\frac{\partial }{\partial x}\left\{D\frac{\partial }{\partial x}{\left[\rho \left(x,t\right)\right]}^{\nu }-F\left(x,t\right)\rho \left(x,t\right)\right\}\;, \end{array}$$

where ν gives the degree of nonlinearity, often used to model diffusion in porous media, where an increase in ν decreases the diffusion. Equation (3) is generally used to describe anomalous behavior often seen in long-range interaction [21], memory effects [22], porous media [23,24], drift terms with contributions not derivable from a potential function [25], and many others (see, for example, Refs. [26,27,28] and references therein). Equation (3) has as solutions non-Gaussian distributions in the absence of external forces. Other situations may consider, for example, a spatial dependence on the diffusion coefficient [29,30]. Both equations satisfy the H-theorem with deep implications for the dependence on this function’s probability density in each case. We are leading to the Boltzmann–Gibbs entropy for Equation (1) and the Tsallis entropy for Equation (3), as discussed in Refs. [31,32,33,34]. Each functional is unique for its respective case and comes from the H-theorem [35,36]. This implies that dH/dt⩾0 and in equilibrium $H_{\mathrm{eq}}=S_{\mathrm{eq}}$, which suggests a relation of the H-theorem with the second law of thermodynamics, as its microscopic counterpart.

Here, we analyze a possible formulation for the H-theorem applied to kinetic equations that are fractional, nonlinear, and have a hyperbolic term, which introduces a finite phase velocity for the relaxation process, such as

(4) $$\begin{array}{c} \tau_{c}\frac{\partial^{2}}{\partial t^{2}}\rho \left(x,t\right)+\int_{0}^{t}dt^{\prime }K_{\gamma }^{\left(1\right)}\left(t-t^{\prime }\right)\frac{\partial }{\partial t^{\prime }}\rho \left(x,t^{\prime }\right)=\frac{\partial }{\partial x}\left\{D\left(\rho \right)\frac{\partial }{\partial x}\rho \left(x,t\right)-F\left(x,t\right)\rho \left(x,t\right)\right\}\;, \end{array}$$

with $K_{\gamma }^{\left(1\right)}\left(t\right)=\delta \left(t\right)+\tau_{\gamma }K_{\gamma }\left(t\right)$, where $\tau_{c}$ is a relaxation time. D(ρ) is a diffusion coefficient with a nonlinear dependence on ρ(x,t). In Equation (4), depending on the choice of $K_{\gamma }\left(t\right)$, we may obtain different integrodifferential operators with singular or non-singular kernels. The Caputo fractional operator can be obtained by considering $K_{\gamma }\left(t\right)$=$t^{-\gamma }$/$\Gamma \left(1-\gamma \right)$ [12], which implies

(5) $$\begin{array}{c} \frac{\partial^{\gamma }}{\partial t^{\gamma }}\rho \left(x,t\right)=\frac{1}{\Gamma \left(1-\gamma \right)}\int_{0}^{t}dt^{\prime }\frac{1}{{\left(t-t^{\prime }\right)}^{\gamma }}\frac{\partial }{\partial t^{\prime }}\rho \left(x,t^{\prime }\right), \end{array}$$

another one is the Fabrizio–Caputo fractional operator, for $K_{\gamma }\left(t\right)$=$k_{\gamma }^{\prime }e^{-\gamma^{\prime }t}$, i.e.,

(6) $$\begin{array}{c} \frac{\partial^{\gamma }}{\partial t^{\gamma }}\rho \left(x,t\right)=k_{\gamma }^{\prime }\int_{0}^{t}dt^{\prime }e^{-\gamma^{\prime }\left(t-t^{\prime }\right)}\frac{\partial }{\partial t^{\prime }}\rho \left(x,t^{\prime }\right)\;, \end{array}$$

or the Atangana–Baleanu fractional operator, for $K_{\gamma }\left(t\right)$=$k_{\gamma }^{\prime }E_{\gamma }\left(-\gamma^{\prime }t^{\gamma }\right)$, given by

(7) $$\begin{array}{c} \frac{\partial^{\gamma }}{\partial t^{\gamma }}\rho \left(x,t\right)=k_{\gamma }^{\prime }\int_{0}^{t}dt^{\prime }E_{\gamma }\left(-\gamma^{\prime }{\left(t-t^{\prime }\right)}^{\gamma }\right)\frac{\partial }{\partial t^{\prime }}\rho \left(x,t^{\prime }\right), \end{array}$$

where $\gamma^{\prime }=\gamma /\left(1-\gamma \right)$, $k_{\gamma }^{\prime }=k_{\gamma }/\left(1-\gamma \right)$, and $k_{\gamma }$ is a normalization constant [14,16,37,38]. It is worth mentioning that the hyperbolic term introduces a finite phase velocity, which is not present in the standard form of the diffusion equation. It introduces a finite velocity of information propagation, which can be related to the finite collision frequency [39,40,41]. Particular cases of Equation (4) arise, for instance, in the analysis of the random walks [42], generalized master equations with memory effects [43], and heat conduction [44]. Nonlinear cases are essential for capturing phenomena such as thermal hysteresis, thermal wave propagation in materials with memory effects, overdamped systems with drag [45], and phase transitions. Other situations can also be connected to the diffusion equations with hyperbolic terms, such as the random Boltzmann-Lorentz gas with Markovian and non-Markovian binary fluctuations [41]. Furthermore, we may also describe situations characterized by different diffusion regimes [46,47] depending on the kernel’s choice or the diffusion coefficient dependence. These scenarios can be found in active intracellular transport [48], systems with long-range interactions [49], particle diffusion in a bacterial bath [50], and motion of organelles and single molecules in living cells [51].

## 2. H-Theorem and Nonlinear Fractional Diffusion-like Equations

We formulate the H-theorem for a general kinetic equation, extending the hyperbolic diffusion equations by considering nonlinear terms such as the one present in Equation (4). For this, we should also note that Equation (4) can be obtained from the combination of the continuity equation

(8) $$\begin{array}{c} \frac{\partial }{\partial t}\rho \left(x,t\right)+\frac{\partial }{\partial x}J\left(x,t\right)=0\;, \end{array}$$

with a suitable choice for the current density J(x,t). In this sense, the current density will be obtained from the H-theorem in combination with a suitable entropic form for the system.

Let us now establish the free energy, i.e., F=U−TS [35,36]. The internal energy and entropy are defined as follows:

(9) $$U\left(t\right)=\int_{-\infty }^{\infty }\rho \left(x,t\right)\phi \left(x\right)dx\;,$$

where the potential, ϕ(x), is related to the external force by the equation $F\left(x\right)=-\partial_{x}\phi \left(x\right)$ and

(10) $$\begin{array}{cc} S(t) & =-k\int_{-\infty }^{\infty }dx\{s\left[\rho \left(x,t\right)\right]+\alpha \left[\rho \left(x,t\right)\right]J^{2}\left(x,t\right)\\ & +\int_{0}^{t}dt^{\prime }\int_{0}^{t^{\prime }}dt^{\prime \prime }K_{\gamma }\left(t^{\prime }-t^{\prime \prime }\right)\vartheta \left[\rho \left(x,t^{\prime }\right)\right]J\left(x,t^{\prime }\right)J\left(x,t^{\prime \prime }\right)\}\;, \end{array}$$

for the system’s entropy, where s[ρ(x,t)], ϑ[ρ(x,t)], α[ρ(x,t)], and J(x,t) will be defined by the H-theorem. Equation (10) extends the one present in [35,36] for a general functional in terms of s(ρ), which may cover different scenarios. The additional terms in Equation (10) will be useful for connecting Equation (4) with an extended thermodynamics [52] and consequently satisfying the H-theorem when memory effects are present in the diffusion process. It is also worth mentioning that in the equilibrium state, J(x,t)=0 and $S=-k\int_{-\infty }^{\infty }dx\;s\left(\rho \right)$ will represent the equilibrium entropy related to the dynamics of the system. In this way, the form of the entropy, that is, how it depends on the probability density, will depend on Equation (4). In the following, we consider the time evolution of the free energy and relate this evolution to the H-theorem to establish the functional probability, which may be connected to Equation (4). In this sense, we have

(11) $$\begin{array}{c} \begin{array}{cc} \frac{d}{dt} & F\left(t\right)=\int_{-\infty }^{\infty }dx\left\{\phi \left(x\right)+k\;T\frac{\partial }{\partial \rho }s\left(\rho \right)+k\;T\left[\frac{\partial }{\partial \rho }\alpha \left(\rho \right)\right]J^{2}\left(x,t\right)\right\}\frac{\partial }{\partial t}\rho \left(x,t\right)\\ & +2k\;T\int_{-\infty }^{\infty }dx\;\alpha \left(\rho \right)J\left(x,t\right)\frac{\partial }{\partial t}J\left(x,t\right)\\ & +k\;T\int_{-\infty }^{\infty }dx\;\vartheta \left(\rho \right)J\left(x,t\right)\int_{0}^{t}dt^{\prime \prime }K_{\gamma }\left(t-t^{\prime \prime }\right)J\left(x,t^{\prime \prime }\right)\;. \end{array} \end{array}$$

By using Equation (8) and performing integration by parts with the boundary condition J(x→∞,t)→0, we can write the previous equation as follows:

(12) $$\begin{array}{c} \begin{array}{cc} \frac{d}{dt} & F\left(t\right)=-\int_{-\infty }^{\infty }dx\left\{\phi \left(x\right)+k\;T\frac{\partial }{\partial \rho }s\left(\rho \right)+k\;T\left[\frac{\partial }{\partial \rho }\alpha \left(\rho \right)\right]J^{2}\left(x,t\right)\right\}\frac{\partial }{\partial x}J\left(x,t\right)\\ & +2\;k\;T\int_{-\infty }^{\infty }dx\;\alpha \left(\rho \right)J\left(x,t\right)\frac{\partial }{\partial t}J\left(x,t\right)+k\;T\int_{-\infty }^{\infty }dx\;\vartheta \left(\rho \right)J\left(x,t\right)\int_{0}^{t}dt^{\prime \prime }K_{\gamma }\left(t-t^{\prime \prime }\right)J\left(x,t^{\prime \prime }\right)\;,\\ & =\int_{-\infty }^{\infty }dxJ\left(x,t\right)\left\{\frac{\partial }{\partial x}\phi \left(x\right)+k\;T\left[\frac{\partial^{2}}{\partial \rho^{2}}s\left(\rho \right)\right]\frac{\partial }{\partial x}\rho \left(x,t\right)\right\}\\ & +k\;T\int_{-\infty }^{\infty }dxJ\left(x,t\right)\frac{\partial }{\partial x}\left[J^{2}\left(x,t\right)\frac{\partial }{\partial \rho }\alpha \left(\rho \right)\right]+2\;k\;T\int_{-\infty }^{\infty }dx\;\alpha \left(\rho \right)J\left(x,t\right)\frac{\partial }{\partial t}J\left(x,t\right)\;\\ & +k\;T\int_{-\infty }^{\infty }dx\;\vartheta \left(\rho \right)J\left(x,t\right)\int_{0}^{t}dt^{\prime \prime }K_{\gamma }\left(t-t^{\prime \prime }\right)J\left(x,t^{\prime \prime }\right)\;,\\ & =\int_{-\infty }^{\infty }dx\frac{J(x,t)}{\rho (x,t)}\left\{\rho \left(x,t\right)\frac{\partial }{\partial x}\phi \left(x\right)+k\;T\rho \left(x,t\right)\left[\frac{\partial^{2}}{\partial \rho^{2}}s\left(\rho \right)\right]\frac{\partial }{\partial x}\rho \left(x,t\right)\right\}\\ & +k\;T\int_{-\infty }^{\infty }dx\frac{J(x,t)}{\rho (x,t)}\rho \left(x,t\right)\left\{\frac{\partial }{\partial x}\left[J^{2}\left(x,t\right)\frac{\partial }{\partial \rho }\alpha \left(\rho \right)\right]+2\;\alpha \left(\rho \right)\frac{\partial }{\partial t}J\left(x,t\right)\right\}\\ & +k\;T\int_{-\infty }^{\infty }dx\frac{J(x,t)}{\rho (x,t)}\rho \left(x,t\right)\left\{\vartheta \left(\rho \right)\int_{0}^{t}dt^{\prime \prime }K_{\gamma }\left(t-t^{\prime \prime }\right)J\left(x,t^{\prime \prime }\right)\right\}\;. \end{array} \end{array}$$

To verify the H-theorem and preserve the negative character of Equation (12), i.e.,

(13) $$\begin{array}{c} \frac{d}{dt}F\left(t\right)\leq 0\;, \end{array}$$

we consider that

(14) $$\begin{array}{c} \begin{array}{cc} J(x,t) & =-\rho \left(x,t\right)\frac{\partial }{\partial x}\phi \left(x\right)-kT\rho \left(x,t\right)\left[\frac{\partial^{2}}{\partial \rho^{2}}s\left(\rho \right)\right]\frac{\partial }{\partial x}\rho \left(x,t\right)\\ & -kT\rho \left(x,t\right)\left\{\frac{\partial }{\partial x}\left[J^{2}\left(x,t\right)\frac{\partial }{\partial \rho }\alpha \left(\rho \right)\right]-2\alpha \left(\rho \right)\frac{\partial }{\partial t}J\left(x,t\right)\right\}\\ & -kT\rho \left(x,t\right)\left\{\vartheta \left(\rho \right)\int_{0}^{t}dt^{\prime \prime }K_{\gamma }\left(t-t^{\prime \prime }\right)J\left(x,t^{\prime \prime }\right)\right\}\;. \end{array} \end{array}$$

Following the discussion present in Refs. [35,52,53], we consider $2kT\rho \alpha \left(\rho \right)=\tau_{c}=const$, $kT\rho \vartheta \left(\rho \right)=\tau_{\gamma }=const$ and neglect the term $J^{2}\left(x,t\right)$ [35] to obtain Equation (4) from the Equations (14) and (8). By substituting Equation (14) in Equation (8), we can obtain Equation (4), i.e.,

(15) $$\begin{array}{c} \tau_{c}\frac{\partial^{2}}{\partial t^{2}}\rho \left(x,t\right)+\int_{0}^{t}dt^{\prime }K_{\gamma }^{\left(1\right)}\left(t-t^{\prime }\right)\frac{\partial }{\partial t^{\prime }}\rho \left(x,t^{\prime }\right)=\frac{\partial }{\partial x}\left\{D\left(\rho \right)\frac{\partial }{\partial x}\rho \left(x,t\right)-F\left(x,t\right)\rho \left(x,t\right)\right\}\;, \end{array}$$

where $K_{\gamma }^{\left(1\right)}\left(t\right)=\delta \left(t\right)+\tau_{\gamma }K_{\gamma }\left(t\right)$ ($\tau_{c}$ is a relaxation time) and the relation to be satisfied:

(16) $$\begin{array}{c} kT\rho \left(x,t\right)\frac{\partial^{2}}{\partial \rho^{2}}s\left(\rho \right)=D\left(\rho \right)\;, \end{array}$$

which connects s(ρ) with D(ρ). This feature implies that different dynamics are related to different forms of entropy (for example, the Tsallis [54,55] and Kaniadakis [56] entropies) to describe the system in consideration. These features can be verified by choosing $D\left(\rho \right)\propto \nu kT\rho^{\nu -1}$, which yields

(17) $$\begin{array}{c} s\left(\rho \right)=\frac{1}{\nu -1}\left(\rho^{\nu }-\rho \right)\;, \end{array}$$

which is essentially connected to the Tsallis entropy [11,57], i.e.,

(18) $$S_{T}\left(t\right)=-\frac{k}{\nu -1}\int_{-\infty }^{\infty }dx\left[\rho^{\nu }\left(x,t\right)-\rho \left(x,t\right)\right]\;.$$

Note that the standard form of the Boltzmann–Gibbs entropy is recovered when taking the limit as ν→1, i.e.,

(19) $$S_{BG}\left(t\right)=-k\int_{-\infty }^{\infty }dx\rho \left(x,t\right)\ln\rho \left(x,t\right)\;,$$

and Equation (4) may be related to the fractional diffusion equation of distributed order [58]. Other forms of entropy imply different choices for dependence present on the diffusion coefficient, D(ρ). These scenarios may allow us to consider the presence of different regimes of diffusion, which can be obtained by considering, for example, $D\left(\rho \right)=D_{1}+\nu D_{\nu }\rho^{\nu -1}$ with $D_{1}\propto T$ and $D_{\nu }\propto T$. This scenario implies that s(ρ) comprises two different entropic forms, i.e.,

(20) $$\begin{array}{c} s\left(\rho \right)=\rho \ln\rho +\frac{1}{\nu -1}\left(\rho^{\nu }-\rho \right)\;, \end{array}$$

one is connected to the linear term and the other to the nonlinear term.

### 2.1. Some Solutions

Let us now consider the solutions of Equation (15) for some cases. We start with the stationary case where the kernel $K_{\gamma }\left(t\right)$ is a power law. The solution for this case is obtained by considering t→∞ for an external force connected with a potential with at least one minimum. In this case, Equation (15) can be simplified and yields the following equation:

(21) $$\begin{array}{c} D\left(\rho_{st}\right)\frac{\partial }{\partial x}\rho_{st}\left(x\right)-F\left(x\right)\rho_{st}\left(x\right)=0\;, \end{array}$$

where $\rho \left(x,t\to \infty \right)=\rho_{st}\left(x\right)$. For the case $D\left(\rho_{st}\right)=\nu kT\rho_{st}^{\nu -1}$, it results in

(22) $$\begin{array}{c} \nu kT\rho_{st}^{\nu -1}\frac{\partial }{\partial x}\rho_{st}\left(x\right)-F\left(x\right)\rho_{st}\left(x\right)=0\;, \end{array}$$

and, consequently,

(23) $$\begin{array}{c} \rho \left(x,t\right)=\frac{1}{Z}\exp_{q}\left[-\frac{\beta }{\nu kT}\phi \left(x\right)\right]\;, \end{array}$$

with $Z^{1-\nu }\beta =1$ and q=2−ν. The above function is the *q*-exponential, and it is defined as follows:

(24) $$\begin{array}{c} \exp_{q}\left[x\right]=\left\{\begin{array}{cc} {\left[1+(1-q)x\right]}^{\frac{1}{1-q}} & \;,x\geq 1/(q-1)\\ 0 & \;,x<1/(q-1) \end{array}\right.\;.\; \end{array}$$

It is worth mentioning that the *q*-exponential naturally emerges from the Tsallis framework.

The solution can be found using standard calculation techniques for the linear case, i.e., ν=1. In particular, for the external force $F\left(x,t\right)=-k_{f}x$, where $k_{f}$ is a constant, it is possible to obtain the solution using the eigenfunctions of the spatial operator related to Equation (4), i.e.,

(25) $$\begin{array}{c} \rho \left(x,t\right)=\sum_{n=0}^{\infty }C_{n}\left(t\right)\psi_{n}\left(x\right) \end{array}$$

with

(26) $$\begin{array}{c} \psi_{n}\left(x\right)=\sqrt{\frac{2D}{\sqrt{\pi }k_{f}}}\frac{e^{-\frac{k_{f}}{2D}x^{2}}}{\sqrt{2^{n}\Gamma \left(1+n\right)}}H_{n}\left(\sqrt{\frac{k_{f}}{2D}}x\right)\;, \end{array}$$

where $H_{n}\left(x\right)$ are the Hermite polynomials [59]. By using the orthogonality of the eigenfunctions, we can obtain an equation for the time-dependent functions $C_{n}\left(t\right)$ and show that it is given by

(27) $$\begin{array}{c} \tau_{c}\frac{\partial^{2}}{\partial t^{2}}C_{n}\left(t\right)+\int_{0}^{t}dt^{\prime }K_{\gamma }\left(t-t^{\prime }\right)\frac{\partial }{\partial t^{\prime }}C_{n}\left(t^{\prime }\right)+\frac{\partial }{\partial t}C_{n}\left(t\right)=-\lambda_{n}DC_{n}\left(t\right)\;, \end{array}$$

with $\lambda_{n}=nk_{f}$ and, for simplicity, $\tau_{\gamma }=1$. The solution of Equation (27) for an arbitrary initial condition, ρ(x,0)=φ(x) can be found by using the Laplace transform ($L\left\{\rho (x,t);s\right\}=\hat{\rho }\left(x,s\right)$ and $L^{-1}\left\{\hat{\rho }\left(x,s\right);t\right\}=\rho \left(x,t\right)$), and it is given by

(28) $$\begin{array}{c} C_{n}\left(t\right)=\Phi_{n}\left(t\right)\int_{-\infty }^{\infty }dx^{\prime }\varphi \left(x^{\prime }\right)\psi_{n}\left(x^{\prime }\right) \end{array}$$

with

(29) $$\begin{array}{c} \Phi_{n}\left(t\right)=L^{-1}\left\{\frac{1+s\tau_{c}+{\hat{K}}_{\gamma }\left(s\right)}{s^{2}\tau_{c}+s+s{\hat{K}}_{\gamma }\left(s\right)+\lambda_{n}D};t\right\}\;. \end{array}$$

The inverse of the Laplace transform can be found in terms of the convolution integrals,

(30) $$\begin{array}{c} \begin{array}{c} \Phi_{n}\left(t\right)=\Xi_{n}\left(t\right)+\sum_{j=1}^{\infty }{\left(-1\right)}^{j}\int_{0}^{t}dt_{j}\Upsilon \left(t-t_{j}\right)\cdots \int_{0}^{t_{2}}dt_{1}\Upsilon \left(t_{2}-t_{1}\right)\Xi_{n}\left(t_{1}\right)\;, \end{array} \end{array}$$

(31) $$\begin{array}{c} \Xi_{n}\left(t\right)=\frac{e^{-\frac{t}{2\tau_{c}}}}{2\Delta_{n}^{\left(1\right)}}\left\{\left(\Delta_{n}^{\left(1\right)}-1\right)e^{-\frac{t\Delta_{n}^{\left(1\right)}}{2\tau_{c}}}\left(\Delta_{n}^{\left(1\right)}+1\right)e^{\frac{t\Delta_{n}^{\left(1\right)}}{2\tau_{c}}}\right\}+\Upsilon_{n}\left(t\right) \end{array}$$

with $\Delta_{n}^{\left(1\right)}=\sqrt{1-4D\lambda_{n}\tau_{c}}$ and

(32) $$\begin{array}{c} \Upsilon_{n}\left(t\right)=\int_{0}^{t}dt^{\prime }K_{\gamma }\left(t-t^{\prime }\right)\frac{2\tau_{c}e^{-\frac{t^{\prime }}{2\tau_{c}}}}{\sqrt{1-4D\lambda_{n}\tau_{c}}}\sinh\left(\frac{t^{\prime }}{2\tau_{c}}\sqrt{1-4D\lambda_{n}\tau_{c}}\right)\;. \end{array}$$

For some particular choices of $K_{\gamma }\left(t\right)$ with $\tau_{c}=0$, it is possible to simplify the previous equation. For example, for $\tau_{c}=0$ and $K_{\gamma }\left(t\right)=k_{\gamma }t^{-\gamma }/\Gamma \left(1-\gamma \right)$, we have

(33) $$\begin{array}{c} \Phi_{n}\left(t\right)=\sum_{j=0}^{\infty }\frac{{\left(-k_{\gamma }t^{\gamma }\right)}^{j}}{\Gamma (1+j)}\left\{E_{1,j\gamma }^{\left(j\right)}\left(-\lambda_{n}Dt\right)+k_{\gamma }t^{\gamma }E_{1,(1+j)\gamma }^{\left(j\right)}\left(-\lambda_{n}Dt\right)\right\} \end{array}$$

with

(34) $$\begin{array}{c} E_{\alpha ,\beta }^{\left(n\right)}\left(t\right)=\frac{d^{n}}{dt^{n}}E_{\alpha ,\beta }\left(t\right)\;, \end{array}$$

where $E_{\alpha ,\beta }\left(t\right)$ is a generalized Mittag–Leffler function [60]. Equation (33) can be written in terms of the H Fox function by using the fact that $E_{\gamma ,\beta }^{\left(n\right)}\left(x\right)=n!\;E_{\gamma ,\beta +\gamma n}^{n+1}\left(x\right)$, n∈N and

(35) $$\begin{array}{c} E_{\gamma ,\beta }^{\delta }\left(x\right)=\sum_{k=0}^{\infty }\frac{{\left(\delta \right)}_{k}}{\Gamma (\gamma k+\beta )}\frac{x^{k}}{k!}=\frac{1}{\delta }H_{1,2}^{1,1}[x{|}_{\left(0,1),(1-\beta ,\gamma \right)}^{\left(1-\delta ,1\right)}]\;, \end{array}$$

where $E_{\gamma ,\beta }^{\delta }\left(x\right)$ is the three-parameter Mittag–Leffler function. By using these results, we have

(36) $$\begin{array}{c} \Phi_{n}\left(t\right)=\sum_{j=0}^{\infty }\frac{{\left(-k_{\gamma }t^{\gamma }\right)}^{j}}{1+j}\left\{H_{1,2}^{1,1}[-\lambda_{n}Dt{|}_{\left(0,1),(1-j\gamma ,1\right)}^{\left(-j,1\right)}]+k_{\gamma }t^{\gamma }H_{1,2}^{1,1}[-\lambda_{n}Dt{|}_{\left(0,1),(1-(1+j)\gamma ,1\right)}^{\left(-j,1\right)}]\right\}. \end{array}$$

For the kernel $K_{\gamma }\left(t\right)=k_{\gamma }^{\prime }e^{-\gamma^{\prime }t}$ ($k_{\gamma }^{\prime }=k_{\gamma }/\left(1-\gamma \right)$ and $\gamma^{\prime }=\gamma /\left(1-\gamma \right)$) with $\tau_{c}=0$, we have

(37) $$\begin{array}{c} \begin{array}{c} \Phi_{n}\left(t\right)=\frac{1}{2\Delta_{n}^{\left(2\right)}}e^{-\frac{\gamma^{\prime }t}{2}\mu_{n}}\left\{\left(\Delta_{n}^{\left(2\right)}-\sigma_{n}\right)e^{\frac{\gamma^{\prime }t\Delta_{n}^{\left(2\right)}}{2}}+\left(\Delta_{n}^{\left(2\right)}+\sigma_{n}\right)e^{-\frac{\gamma^{\prime }t\Delta_{n}^{\left(2\right)}}{2}}\right\}\;. \end{array} \end{array}$$

with $\Delta_{n}^{\left(2\right)}=\sqrt{\left({\left(D\lambda +k_{\gamma }^{\prime }\right)}^{2}/\gamma^{\prime }-2D\lambda +2k_{\gamma }^{\prime }\right)/\gamma^{\prime }+1}$, $\sigma_{n}=D\lambda_{n}/\gamma^{\prime }-k_{\gamma }^{\prime }/\gamma^{\prime }-1$, and $\mu_{n}=D\lambda_{n}/\gamma^{\prime }+k_{\gamma }^{\prime }/\gamma^{\prime }+1$. By using the previous results, we can write Equation (25) as follows:

(38) $$\begin{array}{c} \rho \left(x,t\right)=\int_{-\infty }^{\infty }dx^{\prime }\varphi \left(x^{\prime }\right)\sum_{n=0}^{\infty }\Phi_{n}\left(t\right)\psi_{n}\left(x^{\prime }\right)\psi_{n}\left(x\right)\;. \end{array}$$

Now, we perform some numerical analysis on the solutions of Equation (4) with $D\left(\rho \right)=\nu D\rho^{\nu -1}\left(x,t\right)$ by using the explicit method [61] to obtain the time–space evolution of the equation for the nonlinear fractional equation of distributed order. It is worth mentioning that this numerical solution does not converge for all sets of parameters. The numerical solution was obtained by considering the following discretized equation connected to Equation (4):

(39) $$\begin{array}{c} \rho_{i,j+1}=\beta_{2}\rho_{i,j}-\frac{\tau }{h_{t}^{2}\beta_{1}}\rho_{i,j-1}+\frac{D}{h_{x}^{2}\beta_{1}}\Omega \left(\nu \right)+\frac{k_{f}}{\beta_{1}}F\left(\rho_{i,j}\right)-\frac{1}{\beta_{1}}M\left(\rho_{i,j}\right) \end{array}$$

with

(40) $$\begin{array}{c} \begin{array}{cc} \Omega (\nu ) & =\left(\rho_{i+1,j}^{\nu }-2\rho_{i,j}^{\nu }+\rho_{i-1,j}^{\nu }\right)\;\;,\;\;F\left(\rho_{i,j}\right)=\left(\rho_{ij}+i\frac{\rho_{i+1,j}-\rho_{i-1,j}}{2}\right)\;,\\ M(\rho_{i,j}) & =\sum_{j^{\prime }}K_{\gamma }\left(j,j^{\prime }\right)\left(\rho_{i,j^{\prime }+1}-\rho_{i,j^{\prime }}\right)\;\;,\;\;\beta_{1}=\frac{\tau }{h_{t}^{2}}+\frac{1}{h_{t}}\;,\;\mathrm{and}\;\beta_{2}=\frac{1}{\beta_{1}}\left(\frac{2\tau }{h_{t}^{2}}+\frac{1}{h_{t}}\right)\;. \end{array} \end{array}$$

**Figure 1:**
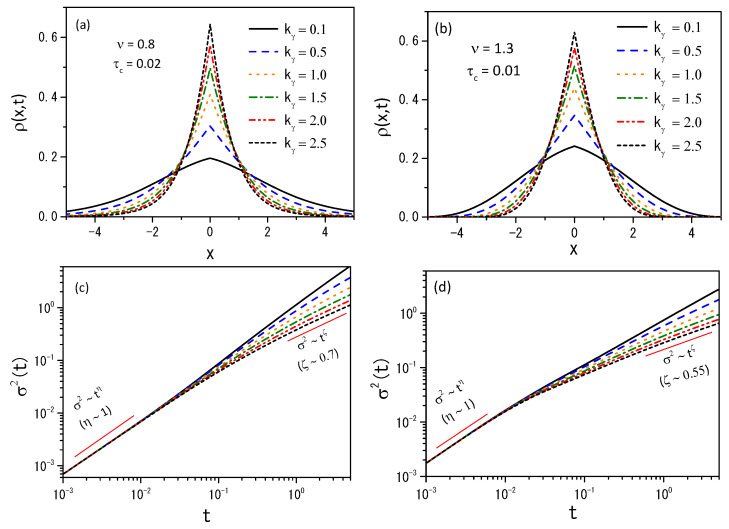
(**a**,**b**) show the behavior of Equation (15) with the probability density distribution for t=4.9 with $h_{x}=0.15$, $h_{t}=0.01$, in the absence of external forces, for ν=0.8 with $\tau_{c}=0.02$ and ν=1.3 with $\tau_{c}=0.01$. (**c**,**d**) show the mean square displacement $\sigma^{2}\left(t\right)=\langle {\left(x-\langle x\rangle \right)}^{2}\rangle$. We consider $K_{\gamma }\left(t\right)=k_{\gamma }t^{-\gamma }/\Gamma \left(1-\gamma \right)$, γ=0.5, $D\left(\rho \right)=\nu D\rho^{\nu -1}$, $\tau_{\gamma }=1$, D=0.5, and different values of $k_{\gamma }$. We also added straight lines to highlight the different behaviors present in the system during the time evolution.

**Figure 2:**
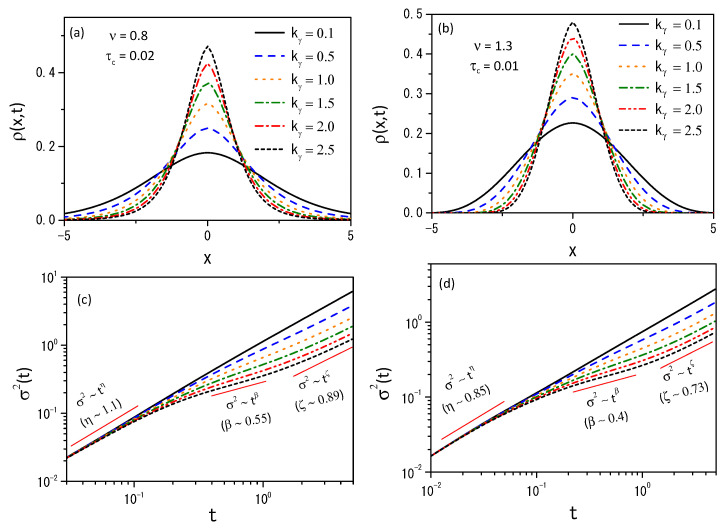
(**a**,**b**) show the behavior of Equation (15) with the probability density distribution at t=4.9 with $h_{x}=0.15$, $h_{t}=0.01$, in the absence of external forces, for ν=0.8 with $\tau_{c}=0.02$ and ν=1.3 with $\tau_{c}=0.01$. (**c**,**d**) show the mean square displacement $\sigma^{2}\left(t\right)=\langle {\left(x-\langle x\rangle \right)}^{2}\rangle$. We consider $K_{\gamma }\left(t\right)=k_{\gamma }^{\prime }e^{-\gamma^{\prime }t}$, γ=0.5, $D\left(\rho \right)=\nu D\rho^{\nu -1}$, $\tau_{\gamma }=1$, D=0.5 and different values of $k_{\gamma }$. We also added straight lines to highlight the different behaviors present in the system during the time evolution.

**Figure 3:**
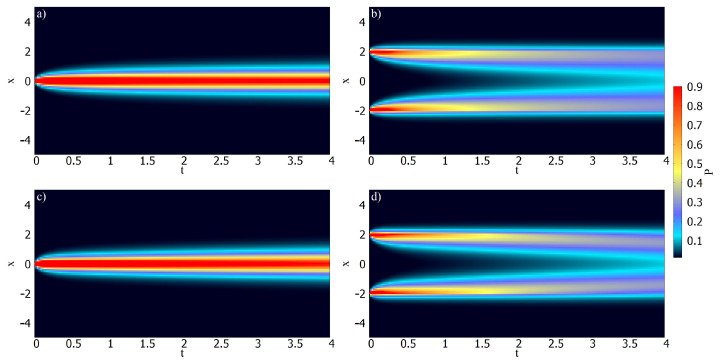
Probability density maps for a pair of initial conditions with $h_{x}=0.10$, $h_{t}=0.01$. In (**a**,**b**), the kernel $K_{\gamma }=k_{\gamma }t^{-\gamma }/\Gamma \left(1-\gamma \right)$ was used and in (**c**,**d**), the kernel $K_{\gamma }=k_{\gamma }^{\prime }e^{-\gamma^{\prime }t}$ was used. For simplicity, we consider ν=0.7, D=0.5, $\tau_{\gamma }=1$, $\tau_{c}=0$, $k_{f}=0.5$, and γ=0.5 for all systems. Note that $K_{\gamma }\left(t\right)$ governed by a power-law is less diffusive than $K_{\gamma }$ governed by an exponential.

The numerical analysis was carried out with the Caputo [12], $K_{\gamma }\left(t\right)=k_{\gamma }t^{-\gamma }/\Gamma \left(1-\gamma \right)$, and Caputo–Fabrizio [15], $K_{\gamma }\left(t\right)=k_{\gamma }^{\prime }e^{-\gamma^{\prime }t}$, kernels for γ=1/2. In Figure 1 and Figure 2, we show trends for the distributions and the mean square displacement for both kernels with ν=0.8, and ν=1.3. They also show different diffusion regimes for the Caputo and Caputo–Fabrizio kernels. Figure 3 shows the diffusion process for Equation (4) with ν=0.7 for two initial conditions ρ(x,0)=δ(x) with $k_{f}=0.5$ for both kernels in absence of the hyperbolic term.

For more numerical results and the deduction of Equation (39), see Appendix A.

### 2.2. Entropy Production

We can examine the entropy production associated with Equation (10) by looking at the dynamics of ρ(x,t) given by Equation (15). Differentiating Equation (10) with respect to time and performing some integration by parts (with J(x→±∞,t)), we obtain that

(41) $$\begin{array}{c} \begin{array}{cc} \frac{d}{dt}S\left(t\right) & =-k\int_{-\infty }^{\infty }dx\left\{\frac{\partial }{\partial \rho }s\left(\rho \right)+\left[\frac{\partial }{\partial \rho }\alpha \left(\rho \right)\right]J^{2}\left(x,t\right)\right\}\frac{\partial }{\partial t}\rho \left(x,t\right)\\ & -\int_{-\infty }^{\infty }dx\alpha \left(\rho \right)J\left(x,t\right)\frac{\partial }{\partial t}J\left(x,t\right)-\int_{-\infty }^{\infty }dx\vartheta \left(\rho \right)J\left(x,t\right)\int_{0}^{t}dt^{\prime \prime }K_{\gamma }\left(t-t^{\prime \prime }\right)J\left(x,t^{\prime \prime }\right)\\ & =k\int_{-\infty }^{\infty }dx\left\{\frac{\partial }{\partial \rho }s\left(\rho \right)+\left[\frac{\partial }{\partial \rho }\alpha \left(\rho \right)\right]J^{2}\left(x,t\right)\right\}\frac{\partial }{\partial x}J\left(x,t\right)\\ & -k\int_{-\infty }^{\infty }dx\alpha \left(\rho \right)J\left(x,t\right)\frac{\partial }{\partial t}J\left(x,t\right)-k\int_{-\infty }^{\infty }dx\vartheta \left(\rho \right)J\left(x,t\right)\int_{0}^{t}dt^{\prime \prime }K_{\gamma }\left(t-t^{\prime \prime }\right)J\left(x,t^{\prime \prime }\right)\\ & =-k\int_{-\infty }^{\infty }dx\left\{\frac{\partial^{2}}{\partial \rho^{2}}s\left(\rho \right)\frac{\partial }{\partial x}\rho \left(x,t\right)+\frac{\partial }{\partial x}\left[\left(\frac{\partial }{\partial \rho }\alpha \left(\rho \right)\right)J^{2}\left(x,t\right)\right]\right\}J\left(x,t\right)\\ & -k\int_{-\infty }^{\infty }dx\alpha \left(\rho \right)J\left(x,t\right)\frac{\partial }{\partial t}J\left(x,t\right)-k\int_{-\infty }^{\infty }dx\vartheta \left(\rho \right)J\left(x,t\right)\int_{0}^{t}dt^{\prime \prime }K_{\gamma }\left(t-t^{\prime \prime }\right)J\left(x,t^{\prime \prime }\right)\;. \end{array} \end{array}$$

Now, by utilizing the equations from the H-theorem,

(42) $$\begin{array}{c} kT\rho \frac{\partial^{2}}{\partial \rho^{2}}s\left(\rho \right)=D\left(\rho \right)\;, \end{array}$$

which connects s(ρ) with D(ρ) and

(43) $$\begin{array}{c} \begin{array}{cc} J(x,t) & =-\rho \left(x,t\right)\frac{\partial }{\partial x}\phi \left(x\right)-kT\rho \left(x,t\right)\left[\frac{\partial^{2}}{\partial \rho^{2}}s\left(\rho \right)\right]\frac{\partial }{\partial x}\rho \left(x,t\right)\\ & -kT\rho \left(x,t\right)\left\{\frac{\partial }{\partial x}\left[J^{2}\left(x,t\right)\frac{\partial }{\partial \rho }\alpha \left(\rho \right)\right]-2\alpha \left(\rho \right)\frac{\partial }{\partial t}J\left(x,t\right)\right\}\\ & +kT\rho \left(x,t\right)\left\{\vartheta \left(\rho \right)\int_{0}^{t}dt^{\prime \prime }K_{\gamma }\left(t-t^{\prime \prime }\right)J\left(x,t^{\prime \prime }\right)\right\}\;. \end{array} \end{array}$$

We obtain that

(44) $$\begin{array}{c} \frac{d}{dt}S\left(t\right)=-\frac{1}{T}\int_{-\infty }^{\infty }dxF\left(x\right)J\left(x,t\right)+\frac{1}{T}\int_{-\infty }^{\infty }dx\frac{J^{2}\left(x,t\right)}{\rho (x,t)}\;. \end{array}$$

Equation (41) can also be written as follows:

(45) $$\begin{array}{c} \frac{d}{dt}S=\Pi -\Phi \;, \end{array}$$

where

(46) $$\begin{array}{c} \Phi =\frac{1}{T}\int_{-\infty }^{\infty }dxF\left(x\right)J\left(x,t\right), \end{array}$$

and the entropy-production term:

(47) $$\begin{array}{c} \Pi =\frac{1}{T}\int_{-\infty }^{\infty }dx\frac{J^{2}\left(x,t\right)}{\rho (x,t)}\;. \end{array}$$

Since *T* and ρ(x,t) are positive, the desired result is Π≥0. This result for the entropy production, given by Equation (44) and, thus, Equation (45), can also be confirmed for any entropy condition.

We performed some numerical calculations using the previous results for the entropy production where $h_{x}$ and $h_{t}$ are increments in position and time, respectively. We perform the numerical simulation via the continuity Equation (8), and $\rho_{i,j}$ obtained via Equation (39), which, after the discretization process, yields:

(48) $$\begin{array}{c} \begin{array}{cc} J_{i+1,j} & =-\frac{\rho_{i,j+1}-\rho_{i,j}}{h_{t}}+J_{i,j}\;,\\ J_{0,j} & =\int_{0}^{\infty }dx\frac{\partial }{\partial t}\rho \left(x,t\right)\approx \frac{h_{x}}{h_{t}}\sum_{i}\left(\rho_{i,j+1}-\rho_{i,j}\right)\;. \end{array} \end{array}$$

Assuming the initial condition ρ(x,0)=δ(x), we have that $J_{i,j}=J_{-i,j}$, and the entropy, in the absence of external forces, can be evaluated by using the following equation:

(49) $$\begin{array}{c} \begin{array}{cc} T\frac{d}{dt}S & \approx h_{x}\sum_{i}\frac{J_{i,j}^{2}}{\rho_{i,j}}={S^{\prime }}_{j}\\ TS_{j} & =h_{t}\sum_{j^{\prime }=0}^{j^{\prime }=j}{S^{\prime }}_{j^{\prime }}\;. \end{array} \end{array}$$

**Figure 4:**
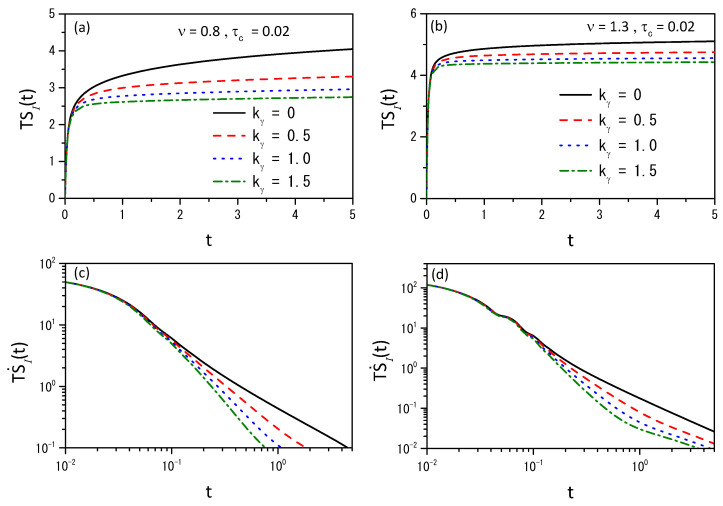
(**a**,**b**) show the behavior of the entropy and (**c**,**d**) show the behavior of Equation (44) for ν=0.8 and ν=1.3 with γ=0.5, $D\left(\rho \right)=\nu D\rho^{\nu -1}$, D=0.5, $\tau_{\gamma }=1$, and different values of $k_{\gamma }$. We considered, for simplicity, ρ(x,0)=δ(x) for the initial condition.

**Figure 5:**
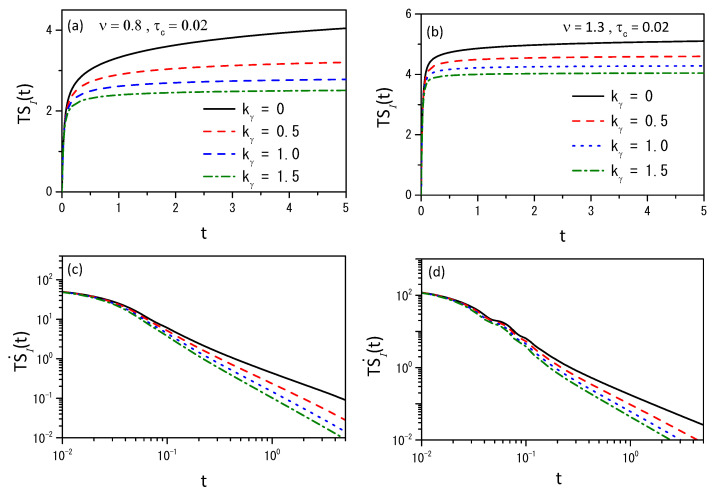
(**a**,**b**) show the behavior of entropy for the power-law kernel, i.e., $K_{\gamma }\left(t\right)=k_{\gamma }t^{-\gamma }/\Gamma \left(1-\gamma \right)$, in the absence of external forces, for ν=0.8 and ν=1.3 with $\tau_{c}=0.02$. (**c**,**d**) show the behavior of Equation (44) for ν=0.8 and ν=1.3. We consider $h_{x}=0.15$, $h_{t}=0.01$, γ=0.5, $D\left(\rho \right)=\nu D\rho^{\nu -1}$, D=0.5, $\tau_{\gamma }=1$, and different values of $k_{\gamma }$. We considered, for simplicity, ρ(x,0)=δ(x) for the initial condition.

**Figure 6:**
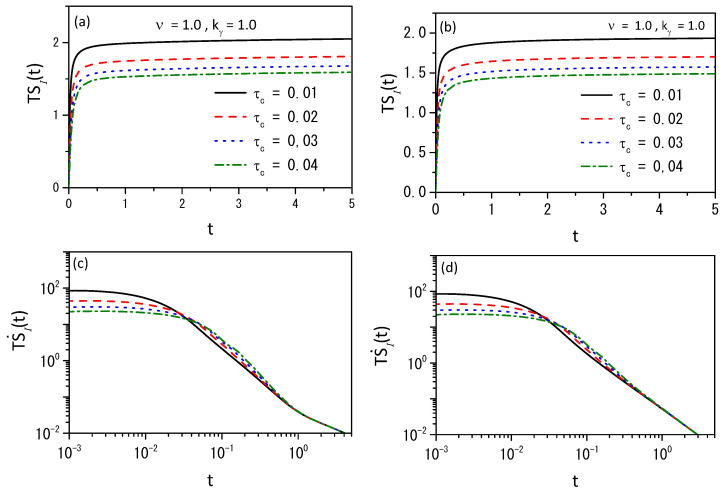
This figure shows the behavior of entropy and Equation (44) for the exponential $K_{\gamma }\left(t\right)=k_{\gamma }^{\prime }e^{-\gamma^{\prime }t}$ (**a**,**c**) and power-law $K_{\gamma }\left(t\right)=k_{\gamma }t^{-\gamma }/\Gamma \left(1-\gamma \right)$, (**b**,**d**) kernels in the absence of external forces. We consider $h_{x}=0.15$, $h_{t}=0.01$, γ=0.5, $D\left(\rho \right)=\nu D\rho^{\nu -1}$, D=0.25, $\tau_{\gamma }=1$, and different values of $\tau_{c}$. We considered, for simplicity, ρ(x,0)=δ(x) for the initial condition.

Figure 4, Figure 5 and Figure 6 illustrate the entropy and the entropy production for different scenarios to show that different behaviors can be obtained connected to the different choices of the kernels.

## 3. Conclusions

Considering the memory effect, we have investigated the H-theorem for nonlinear fractional diffusion equations, which may present different forms of nonlinearity on the diffusive term. We followed the approaches employed in Ref. [36] by extending the entropy, an arbitrary probability density function, to cover different scenarios. Consequently, the entropy results from the H-theorem may have properties different from the usual, as pointed out in Refs. [33,62,63]. The nonlinear hyperbolic diffusion-like equations emerging from this approach have been analyzed from both analytical and numerical points of view. Analytically, we found the solutions for the linear case by expanding in terms of the eigenfunctions. Numerically, we studied the solutions of Equation (4) by using its discretized form, given by Equation (39), to investigate the dynamics of the nonlinear case. In particular, we considered exponential and power-law kernels to investigate the different dynamics and their relaxation processes; see Figure 1, Figure 2 and Figure 3. We have also analyzed the entropy production for different scenarios, as shown in Figure 4, Figure 5 and Figure 6. Finally, we hope the results presented here may be useful in discussing nonlinear hyperbolic diffusion equations, the H-theorem, and, consequently, the entropies. 

## Figures and Tables

**Figure A1:**
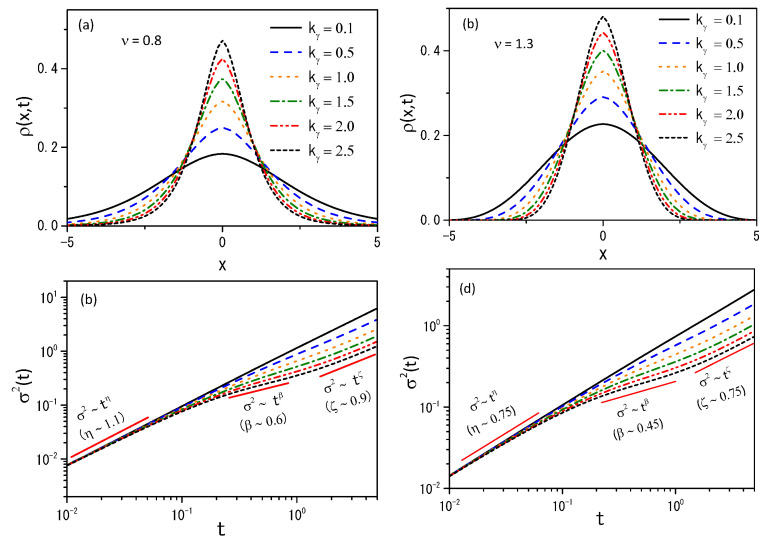
(**a**,**b**) illustrate the behavior of Equation (15) in the absence of external forces. (**c**,**d**) show the mean square displacement $\sigma^{2}\left(t\right)=\langle {\left(x-\langle x\rangle \right)}^{2}\rangle$, for ν=0.8 and ν=1.3. We consider $h_{x}=0.15$, $h_{t}=0.01$, $K_{\gamma }\left(t\right)=k_{\gamma }^{\prime }e^{-\gamma^{\prime }t}$, $\tau_{\gamma }=1$, γ=0.5, $D\left(\rho \right)=\nu D\rho^{\nu -1}$, and different values of $k_{\gamma }$. We also added straight lines to highlight the different behaviors present in the system during the time evolution.

**Figure A2:**
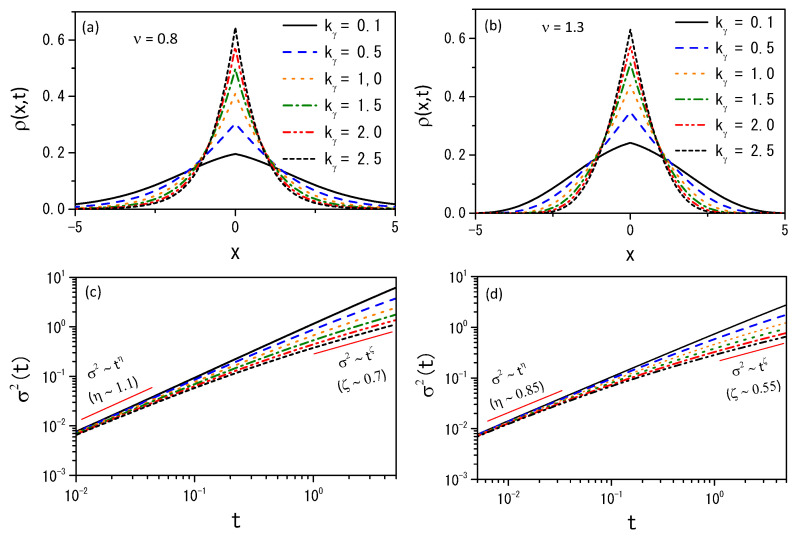
(**a**,**b**) illustrate the behavior of Equation (15) in the absence of external forces. (**c**,**d**) show the mean square displacement $\sigma^{2}\left(t\right)=\langle {\left(x-\langle x\rangle \right)}^{2}\rangle$, for ν=0.8 and ν=1.3. We consider $h_{x}=0.15$, $h_{t}=0.01$, $K_{\gamma }\left(t\right)=k_{\gamma }t^{-\gamma }/\Gamma \left(1-\gamma \right)$, $\tau_{\gamma }=1$, γ=0.5, $D\left(\rho \right)=\nu D\rho^{\nu -1}$, and different values of $k_{\gamma }$. We also added straight lines to highlight the different behaviors present in the system during the time evolution.

**Figure A3:**
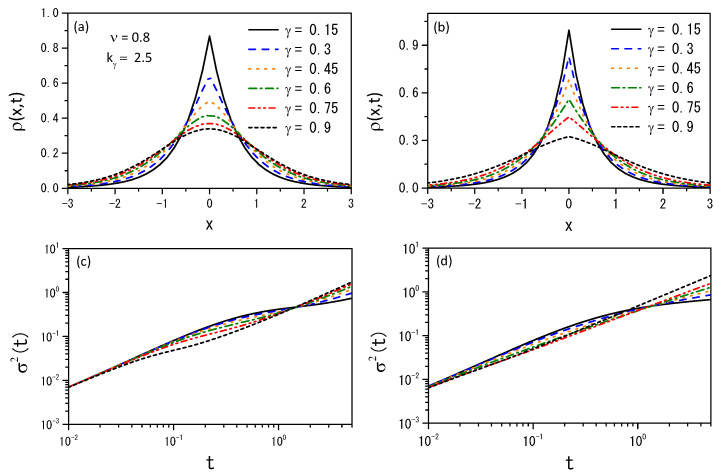
This figure shows the behavior of Equation (15) in the absence of external forces, and the mean square displacement $\sigma^{2}\left(t\right)=\langle {\left(x-\langle x\rangle \right)}^{2}\rangle$, for ν=0.8, $\tau_{\gamma }=1$, $k_{\gamma }=2.5$, and $\tau_{c}=0.02$. We consider $h_{x}=0.15$, $h_{t}=0.01$, $K_{\gamma }\left(t\right)=k_{\gamma }^{\prime }e^{-\gamma^{\prime }t}$ in (**a**,**c**) and $K_{\gamma }\left(t\right)=k_{\gamma }t^{-\gamma }/\Gamma \left(1-\gamma \right)$ in (**b**,**d**), γ=0.5, $D\left(\rho \right)=\nu D\rho^{\nu -1}$, and different values of γ.

## Data Availability

The original contributions presented in the study are included in the article, further inquiries can be directed to the corresponding author.

## Appendix A. Numerical Results

In Appendix A.1 we discuss the steps involved in obtaining Equation (39) from Equation (4) via the explicit method [61]. In Appendix A.2 some numerical results obtained will be discussed.

### Appendix A.1. Explicit Method

Let us start with the nonlinear fractional partial differential equation to be solved:

(A1) $$\begin{array}{c} \begin{array}{cc} \tau_{c}\frac{\partial^{2}}{\partial t^{2}}\rho \left(x,t\right) & +\int_{0}^{t}dt^{\prime }K_{\gamma }\left(t-t^{\prime }\right)\frac{\partial }{\partial t^{\prime }}\rho \left(x,t^{\prime }\right)\\ & +\frac{\partial }{\partial t}\rho \left(x,t\right)=\frac{\partial }{\partial x}\left\{D\frac{\partial }{\partial x}{\left[\rho \left(x,t\right)\right]}^{\nu }+k_{f}x\rho \left(x,t\right)\right\}\;, \end{array} \end{array}$$

where, for simplicity, $\tau_{\gamma }=1$. The first step is to discretize Equation (A1) to a cell $\rho_{i,j}$, where *i* denotes an increment in space of size $h_{x}$ and *j* is an increment in time with size $h_{t}$. We now define three numerical approximations for partial differentiation, starting with the forward rules:

(A2) $$\begin{array}{c} \begin{array}{c} \frac{\partial }{\partial t}\rho \left(x,t\right)\approx \frac{\rho_{i,j+1}-\rho_{i,j}}{h_{t}}\;,\;\frac{\partial }{\partial x}\rho \left(x,t\right)\approx \frac{\rho_{i+1,j}-\rho_{i,j}}{h_{x}}\;, \end{array} \end{array}$$

the backward rules,

(A3) $$\begin{array}{c} \begin{array}{c} \frac{\partial }{\partial t}\rho \left(x,t\right)\approx \frac{\rho_{i,j}-\rho_{i,j-1}}{h_{t}}\;,\;\frac{\partial }{\partial x}\rho \left(x,t\right)\approx \frac{\rho_{i,j}-\rho_{i-1,j}}{h_{x}}\;, \end{array} \end{array}$$

and the centered rule:

(A4) $$\frac{\partial }{\partial x}\rho \left(x,t\right)\approx \frac{\rho_{i+1,j}-\rho_{i-1,j}}{2h_{x}}\;.$$

We now may solve the integral of Equation (A1) using a Riemann sum and applying the forward derivative rule to $\partial_{t^{\prime }}\rho \left(x,t^{\prime }\right)$, assuming the use of the Caputo kernel for didactic purpose:

(A5) $$\begin{array}{c} \begin{array}{cc} K_{\gamma }\left(t-t^{\prime }\right) & =\frac{k_{\gamma }}{\Gamma \left(1/2\right)\sqrt{t-t^{\prime }}}\;,\;K_{\gamma }\left(j-j^{\prime }\right)=\frac{k_{\gamma }}{\Gamma \left(1/2\right)\sqrt{h_{t}\left(j-j^{\prime }\right)}}\;,\;\mathrm{and}\\ \int_{0}^{t}dt^{\prime }K_{\gamma }\left(t-t^{\prime }\right)\frac{\partial }{\partial t^{\prime }}\rho \left(x,t^{\prime }\right) & \approx \frac{1}{\Gamma (1/2)}\sum_{j^{\prime }=0}^{j^{\prime }<j}\frac{h_{t}k_{\gamma }}{\sqrt{h_{t}\left(j-j^{\prime }\right)}}\left(\frac{\rho_{i,j^{\prime }+1}-\rho_{i,j^{\prime }}}{h_{t}}\right)=M\left(\rho_{i,j}\right)\;. \end{array} \end{array}$$

For the temporal derivatives, using the forward derivative rule, we have the following:

(A6) $$\begin{array}{c} \begin{array}{c} \tau_{c}\frac{\partial^{2}}{\partial t^{2}}\rho \left(x,t\right)+\frac{\partial }{\partial t}\rho \left(x,t\right)\approx \tau_{c}\frac{\partial }{\partial t}\left(\frac{\rho_{i,j+1}-\rho_{i,j}}{h_{t}}\right)+\frac{\rho_{i,j+1}-\rho_{i,j}}{h_{t}}\;. \end{array} \end{array}$$

Applying the backward rule to the first term and the forward rule to the second term of the derivative, we have the following:

(A7) $$\begin{array}{c} \begin{array}{cc} \tau_{c}\frac{\partial^{2}}{\partial t^{2}}\rho \left(x,t\right)+\frac{\partial }{\partial t}\rho \left(x,t\right) & \approx \tau_{c}\left(\frac{\left(\rho_{i,j+1}-\rho_{i,j}\right)-\left(\rho_{i,j}-\rho_{i,j-1}\right)}{h_{t}^{2}}\right)+\frac{\rho_{i,j+1}-\rho_{i,j}}{h_{t}}\\ \tau_{c}\frac{\partial^{2}}{\partial t^{2}}\rho \left(x,t\right)+\frac{\partial }{\partial t}\rho \left(x,t\right) & \approx \tau_{c}\left(\frac{\rho_{i,j+1}-2\rho_{i,j}+\rho_{i,j-1}}{h_{t}^{2}}\right)+\frac{\rho_{i,j+1}-\rho_{i,j}}{h_{t}}\;, \end{array} \end{array}$$

which yields the follwoing:

(A8) $$\begin{array}{c} \tau_{c}\frac{\partial^{2}}{\partial t^{2}}\rho \left(x,t\right)+\frac{\partial }{\partial t}\rho \left(x,t\right)\approx \rho_{i,j+1}\left(\frac{\tau_{c}}{h_{t}^{2}}+\frac{1}{h_{t}}\right)-\rho_{i,j}\left(\frac{2\tau_{c}}{h_{t}^{2}}+\frac{1}{h_{t}}\right)+\rho_{i,j-1}\left(\frac{\tau_{c}}{h_{t}^{2}}\right)\;. \end{array}$$

Let $\beta_{1}=\tau /h_{t}^{2}+1/h_{t}$, and the temporal part becomes

(A9) $$\begin{array}{c} \begin{array}{cc} \tau_{c}\frac{\partial^{2}}{\partial t^{2}}\rho \left(x,t\right)+\int_{0}^{t}dt^{\prime }K_{\gamma }\left(t-t^{\prime }\right)\frac{\partial }{\partial t^{\prime }}\rho \left(x,t^{\prime }\right) & +\frac{\partial }{\partial t}\rho \left(x,t\right)\approx \beta_{1}\rho_{i,j+1}\\ & -\rho_{i,j}\left(\frac{2\tau_{c}}{h_{t}^{2}}+\frac{1}{h_{t}}\right)+\rho_{i,j-1}\left(\frac{\tau_{c}}{h_{t}^{2}}\right)+M\left(\rho_{i,j}\right)\;. \end{array} \end{array}$$

Solving the force term and using the centered rule:

(A10) $$\begin{array}{c} \begin{array}{cc} k_{f}\frac{\partial }{\partial x}x\rho \left(x,t\right) & =k_{f}\left(\rho \left(x,t\right)+x\frac{\partial }{\partial x}\rho \left(x,t\right)\right)\;,\\ & \approx k_{f}\left(\rho_{i,j}+ih_{x}\frac{\rho_{i+1,j}-\rho_{i-1,j}}{2h_{x}}\right)\;,\\ & \approx k_{f}\left(u_{i,j}+i\frac{\rho_{i+1,j}-\rho_{i-1,j}}{2}\right)\;. \end{array} \end{array}$$

We now may begin by applying the forward rule to the diffusion term of Equation (A9):

(A11) $$\begin{array}{c} \frac{\partial^{2}}{\partial x^{2}}\rho {\left(x,t\right)}^{\nu }\approx \frac{D}{h_{x}}\frac{\partial }{\partial x}\left(\rho_{i+1,j}^{\nu }-\rho_{i,j}^{\nu }\right)\;. \end{array}$$

Next, we derive again using the backward rule for the first term and the forward rule for the right term:

(A12) $$\begin{array}{c} \begin{array}{cc} \frac{\partial^{2}}{\partial x^{2}}\rho {\left(x,t\right)}^{\nu } & \approx \frac{D}{h_{x}^{2}}\left(\left(\rho_{i+1,j}^{\nu }-\rho_{i,j}^{\nu }\right)-\left(\rho_{i,j}^{\nu }-\rho_{i-1,j}^{\nu }\right)\right)\\ \frac{\partial^{2}}{\partial x^{2}}\rho {\left(x,t\right)}^{\nu } & \approx \frac{D}{h_{x}^{2}}\left(\rho_{i+1,j}^{\nu }-2\rho_{i,j}^{\nu }+\rho_{i-1,j}^{\nu }\right)\;. \end{array} \end{array}$$

Combining and rearranging all previously obtained terms, we get Equation (39):

(A13) $$\begin{array}{c} \begin{array}{cc} \rho_{i,j+1} & =\beta_{2}\rho_{i,j}-\frac{\tau }{\beta_{1}h_{t}^{2}}\rho_{i,j-1}+\frac{D}{h_{x}^{2}\beta_{1}}\Omega \left(\nu \right)+\frac{k_{f}}{\beta_{1}}F\left(\rho_{i,j}\right)-\frac{1}{\beta_{1}}M\left(\rho_{i,j}\right)\;,\\ \Omega (\nu ) & =\left(\rho_{i+1,j}^{\nu }-2\rho_{i,j}^{\nu }+\rho_{i-1,j}^{\nu }\right)\;\;,\;\;F\left(\rho_{i,j}\right)=\left(\rho_{ij}+i\frac{\rho_{i+1,j}-\rho_{i-1,j}}{2}\right)\;,\\ M(\rho_{i,j}) & =\frac{1}{\Gamma (1/2)}\sum_{j^{\prime }=0}^{j^{\prime }<j}\frac{k_{\gamma }}{\sqrt{h_{t}\left(j-j^{\prime }\right)}}\left(\rho_{i,j^{\prime }+1}-\rho_{i,j^{\prime }}\right)\;,\\ \beta_{1} & =\frac{\tau }{h_{t}^{2}}+\frac{1}{h_{t}}\;\;,\;\mathrm{and}\;\beta_{2}=\frac{1}{\beta_{1}}\left(\frac{2\tau }{h_{t}^{2}}+\frac{1}{h_{t}}\right)\;. \end{array} \end{array}$$

### Appendix A.2. Numerical Results

Using the results from the previous section, we can perform numerical calculations. In particular, we perform some numerical calculations by using the kernels:

(A14) $$K_{\gamma }\left(t\right)=\frac{k_{\gamma }}{1-\gamma }e^{-\gamma^{\prime }t}\;\;\;\;\;\left(\gamma^{\prime }=\frac{\gamma }{1-\gamma }\right)\;,\;\mathrm{and}\;K_{\gamma }\left(t\right)=\frac{k_{\gamma }t^{-\gamma }}{\Gamma (1-\gamma )}\;,$$

in absence of external forces with $\tau_{c}=0$ (see, Figure A1 and Figure A2).

In the following figure, Figure A3, we consider different values of γ with $\tau_{c}\neq 0$.
